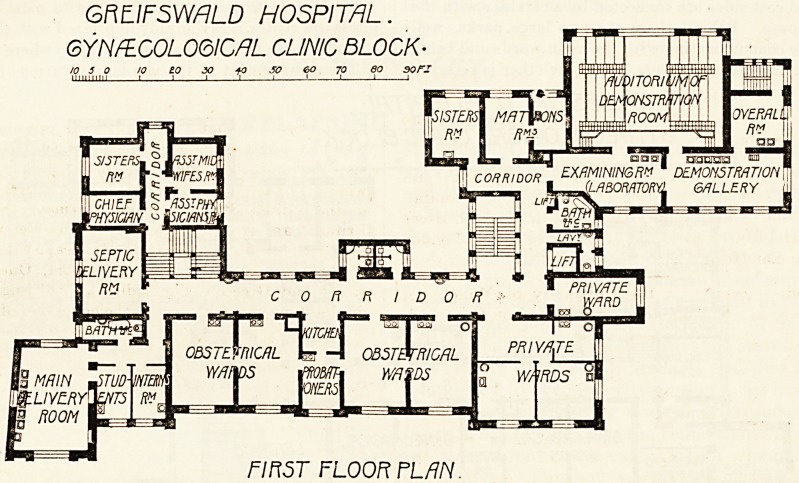# Some Modern Continental Hospitals

**Published:** 1908-09-12

**Authors:** 


### HOSPITAL ADMINISTRATION.

### CONSTRUCTION AND ECONOMICS.

### SOME MODERN CONTINENTAL HOSPITALS.

THE NEW GYNÆCOLOGICAL CLINIC AT GREIFSWALD.

THE old Frauenklinik at Greifswald, dating back to the year 1878, has recently been rebuilt and modified, so that at present it ranks as one of the best of its class on the Continent. The rebuilding took place under the supervision of Professor Dr. Martin, late director of the clinic, and Herr Ernst Lucht, consulting architect to the Greifswald University, and the result shows very well what can be obtained when the professional head of an institution works in close consultation and agreement with the designer. For the use of the attached plans and diagrams we are indebted to the official account of the reconstruction which appeared in the "Klinischer Jahrbuch, 1908."

The main portion of the old building and the additions on the west and east sides are connected by a corridor with the director's house. Behind these are two large parks, well laid out, one communicating with the main wards and being destined for the use of patients, while the other is reserved as an attachment to the director's house. On the east side is the lodging for the officials, flanked by a well-arranged economic department, containing the steam laundry. On the north side lies the city part, separated from the hospital by a high wall on the inner side of which are three small buildings, one used as a coal-house, the middle one for refuse and waste and the third as stable and kennels for experimental purposes. The main entrance faces east, and is reached through a small circular park.

The main object which has been kept in view in the new building is to provide adequate accommodation for (1) patients, (2) operations, (3) out-patient work, (4) lectures and educational purposes generally, (5) experimental and scientific purposes, and finally for (6) administrative purposes. With that object in view there have been provided several separate entrances, so that students, nurses, and patients' friends are not forced to use the same entrance. The buildings, of red brick with cement facings, are double-storied, and the hospital has been constructed on a modified corridor system, and externally impresses the visitor by its quiet simplicity and harmonious proportions, without forcing admiration on account of merely architectural beauty, as so many of the newer Continental hospitals do. In the basement of the main building are located the large and well-equipped kitchens, sculleries, pantries, and larder, with rooms for the personnel and extra rooms for various purposes. Here too are the machine rooms for providing light and heating, all of which are provided with the latest and most up-to-date apparatus. The lavatory accommodation is excellent and fully adequate. The rooms are well lit and airy, there being no buildings near to obscure the light or interefere with free ventilation. As in most German hospitals the visitor is favourably impressed with the scrupulous cleanliness and order that prevail everywhere.

The arrangement of the wards and various lecture rooms will be readily seen from the accompanying plans. Thus on the ground floor the main feature is the fine operating room for ordinary cases and the equally fine septic operating room. The first promises to be one of the finest operating theatres in Germany. It is efficiently lit by large open windows, while for artificial lighting the Zeiss-krönig lamps, giving a light equal to 32,000 candle-power, are provided, thus furnishing a powerful but diffused illumination. The arrangement by means of which this electric light is reflected into the room, by means of a swinging concave mirror, is interesting. The disadvantage of this method is, of course, the intense heat caused by such strong reflection, but this is largely obviated by the excellent free ventilation. The lamps are, of course, outside the theatre itself.

The floor is of terrazzo and the walls are tiled half way up. All corners have been carefully rounded off and the theatre can be easily and effectively cleansed by means of hose pipes fixed to the corridor hydrants. Especially interesting is the fine operating table, by Schille, of Stockholm, after designs by Professor Martin himself. This table presents many remarkable points, and is particularly suitable for abdominal work. Attached to the theatre are the usual sterilising and preparing rooms, including the anæsthetising room, where patients are prepared for lumbar puncture, tropacocaine being the usual anæsthetic used at this clinic. The main gynæcological wards are on this floor, lying to the right of the theatre. They consist of four wards, each accommodating ten patients, and opening on to the main corridor. These wards are roomy and well ventilated, the flooring is of linoleum resting partly on cement and partly (in the older part) on a wood foundation. The ventilation is on a modified central system, the fresh air being first filtered and warmed, and then distributed by means of special ventilation valves, the result being that a more or less equable temperature is maintained in the wards, although they lack the cosy warmth of the open fireplace. In winter the heating is provided for by an extended system of low-pressure steam pipes which run underneath the corridor, and radiators are fixed in every room. The wards are comfortable and free from dust-collecting addenda.

separated from the ordinary gynæcological division, than which it lies on a slightly lower level, is the department for septic gynæcological cases. This department, which is complete in itself, consists of a large septic operating theatre, a bath-room, and two wards, each containing two beds. The theatre is well ventilated and lighted by large windows; artificial light is furnished by several Osmium lamps arranged outside the room, so that, as in the case of the main theatre, the light is diffuse, and dust collection is avoided. This division is almost hermetically sealed off from the other department by means of swing doors, which fit closely into their frames, and the only room close to it is the "dying room" already referred to. As in the main division the wards are lighted mainly from the south side, in part also from the east.

On the first floor are housed the obstetrical patients, the plan being similar to that of the ground floor, with the exception that the portion devoted on the latter to the septic division is here used for rooms for the sister and staff. The main obstetrical wards, four in number, are similar to those for the use of gynæcological patients, and between them, as on the ground floor, is the ward kitchen.

GREIFSWALD HOSPITAL.
GYNÆCOLOGICAL CLINIC BLOCK.

FIRST FLOOR PLAN.

They have a pleasant, attractive appearance, and the large windows give the patients a good view of the park on one side. A praiseworthy feature in the institution is the provision of the "room for moribund patients." Into this room, which is really a small ward, quite separated from the main wards, patients whose condition is deemed hopeless are brought. This arrangement obviates the carrying into the general wards of biers or similar funereal appliances, the appearance of which must necessarily have some influence even upon the strongest-minded patients. Such an arrangement is to be heartily commended, and one can only hope that in the near future it will be imitated generally. It necessarily entails a little extra trouble to the staff, but such trouble is adequately compensated by the advantages it brings. The lavatory accommodation in each ward is well provided for, the basins being large and the flushing system working well. Bath-room and closet accommodation is also liberally provided for. For the transport of patients and visitors an automatic electric lift, with safety arrangements, has been constructed. In the wards care has been taken to keep the floors absolutely free—nothing is fixed or screwed down.

On the same floor and on the same side of it, though quite

In each ward are six beds, arranged between the windows, which face south. The air space is thus more than sufficient, and the wards can be regarded as extravagant even. To the left is the large delivery room, built over the main operating theatre. This has a fine equipment, the arrangement whereby hot or cold water in large quantity can be obtained being especially worthy of notice. In this room the usual midwifery manipulations are carried out, while in the adjoining—though entirely separate—septic delivery room septic cases are delivered. These rooms, like the lower theatres, have terrazzo floors and half-tiled walls. On the east side are several smaller wards for private patients, the so-called "class rooms," which are used for the accommodation of paying patients, who have their own closet and lavatory accommodation. Alongside the main delivery theatre is the students' room and the interns' room, with which, as with the other purely demonstrative and educational parts of the clinic, we deal in the next article. The laundry and administrative block lies, as can be seen from the ground plan, on the west side, the real administrator being, of course, the director, who has his private house in the grounds. The laundry itself is a thoroughly up-to-date institution, but offers nothing specially noteworthy in its internal arrangements. Everywhere fire hydrants are provided, and, in general, it may be stated here that the modern continental hospital appears to be much better protected against an outbreak of fire than are our large hospitals, who still to a great extent rely on inadequate and antiquated hand apparatus and extinguishers. The water supply is obtained from the main city pipes, and stored in large reservoirs so that the institution is, in a sense, independent of the communal mains. Wherever the pipes are in any way exposed efficient precautions have been taken against frost. All the closets are provided with forcible cataract flushing systems. Wards, waiting rooms, and residential quarters are lit by pendant gas mantles, which are found far less trying to the eyes than electric light. This latter is, however, found in the operating rooms, the power being furnished from the city station. In the laundry block every provision has been made for sterilisation and disinfection. The costs of the building to date, inclusive of the director's house and the various educational rooms, may be estimated from the following figures : Cost of the old building, 246,376 marks; costs of added buildings and director's house up to 1884, 80,000 marks (approximate); expenses of constructing east wing (1899-1901), 260,000 marks; cost of construction and rebuilding of western wing and septic division, 150,000 marks. Total cost of old buildings and reconstruction approximately 740,000 marks, or nearly £37,000, which, taking all things into consideration, is a comparatively small sum—approximately £400 per bed—for the construction of what may be classed as one of the finest clinics in Germany.

(*To be continued.*)

## Figures and Tables

**Figure f1:**
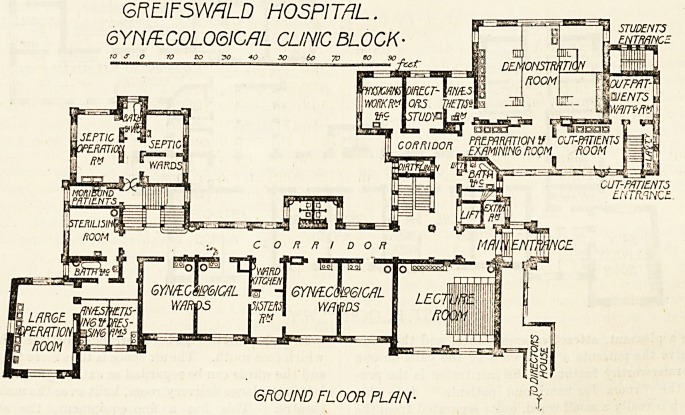


**Figure f2:**